# Supplementary material for: The effects of vessel noise on the communication network of humpback whales
Source: R Soc Open Sci. 2019 Nov 27;6(11):190967. doi: 10.1098/rsos.190967 (PMC6894609; doi:10.1098/rsos.190967)
Supplement: Supplementary Methods [file rsos190967supp1.docx]

**Supplementary Materials and Methods**

**Communication space.** A Generalised Additive Model framework in “R” software with the MRSea (Scott-Hayward et al. 2014) and geepack (Yan and Fine, 2004; Højsgaard et al. 2006) packages for were used model fitting and selection. The response data, *SNR* at the array (termed *rSNR*), ranged from -28 to 37 dB. A frequency (3^rd^ quartile frequency) and duration (3^rd^ quartile duration) measure were considered as one-dimensional smooth terms and an interaction term assessed between broadband noise and signaller distance from the array (by fitting a two-dimensional smooth term as outlined below).

A Complex Region Spatial Smoother (CReSS) (Scott-Hayward et al. 2014) was used to fit the two-dimensional smooth surface to the interaction between noise and distance using a Spatially Adaptive Local Smoothing Algorithm (SALSA; Walker et al. 2011). In short, this analysis procedure is used is for surfaces, where knots, being the sources of flexibility of the surface, can raise or lower the surface. A knot-selection process (SALSA) is used to select the position of the knots, and a smoothing method (CReSS) is used to manipulate the flexibility of the surface based on the relationship between the two variables. The surface is modelled as the sum of basis functions that decay exponentially with distance from a distribution of ‘knots’, with the rate of exponential decay chosen to show local patterns.

First, the two interacting covariates (noise and distance) were scaled and centred, and smooth terms fitted using degree 2 B-splines (Scott-Hayward et al. 2013). Then, a Bayesian Information Criteria was used for selection of number, and location, of ‘knots’. Model selection of covariates used a five-fold Cross-Validation (CV) procedure, where the smaller number indicated a better fitting model. One dimensional covariates (frequency and duration) were also considered and included as smooth terms if significant. The optimal model was rerun in a Generalized Estimating Equation (GEE; Hardin, 2005) with ‘Sound ID’ as the panel structure to account for multiple measurements being made on the same sound. Predictions were made from the final GEE model (after setting significant one-dimensional smooth terms to the mean) and presented in figures. The following figures (Fig. S2 and S3) are the output of these models for wind-dominated (Dunlop, 2018) and vessel (Dunlop, in press) noise. Signal source level was not included in any of the models as a one-dimensional smooth term meaning any source level adjustment by the signaller to noise was included in the output.

| Model structure | Signal type | Sample size | Predictors | *P* value |
| --- | --- | --- | --- | --- |
| *rSNR* ~ s(*NL_bb_* wind, Distance, d.f. = 6) + Soundgp+ 3^rd^ quartile frequency +  3^rd^ quartile time | Low-frequency | 15 | s(*NL_bb_* wind, Distance)  Frequency  Time | <0.0001  <0.0001  <0.0001 |
| *rSNR* ~ s(*NL_bb_* vessel, Distance, d.f. = 5) + Soundgp+ 3^rd^ quartile frequency +  3^rd^ quartile time | Low-frequency | 16 | s(*NL_bb_* vessel, Distance)  Frequency  Time | <0.0001  <0.001  <0.0001 |
| *rSNR* ~ s(*NL_bb_* wind, Distance, d.f. = 6) + Soundgp+ 3^rd^ quartile frequency +  3^rd^ quartile time | High-frequency | 8 | s(*NL_bb_* wind, Distance)  Frequency  Time | <0.0001  <0.0001  <0.0001 |
| *rSNR* ~ s(*NL_bb_* vessel, Distance, d.f. = 5) + Soundgp+ 3^rd^ quartile frequency +  3^rd^ quartile time | High-frequency | 8 | s(*NL_bb_* vessel, Distance)  Frequency  Time | <0.0001  <0.001  <0.0001 |

Table S1. The generalized estimating equation outputs from Dunlop (2018 and in press for wind-dominated and vessel noise respectively) showing the model structure including degrees of freedom (d.f.) for the 2D smooth variable, group sample size, significance of predictor variables.


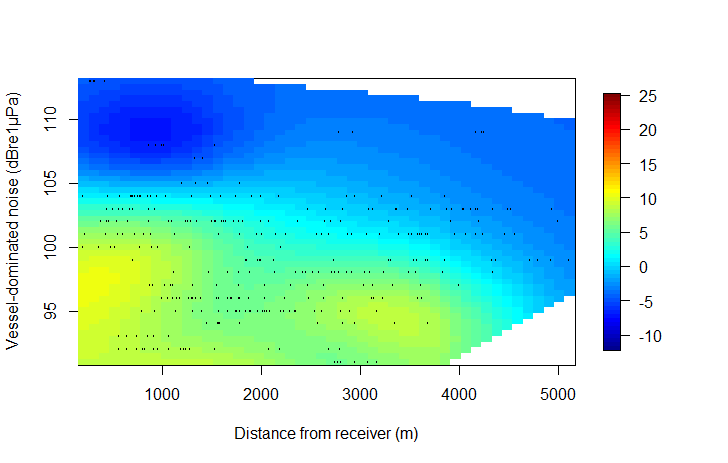


.

**a**

Broadband wind noise (dB *re* 1 µPa)


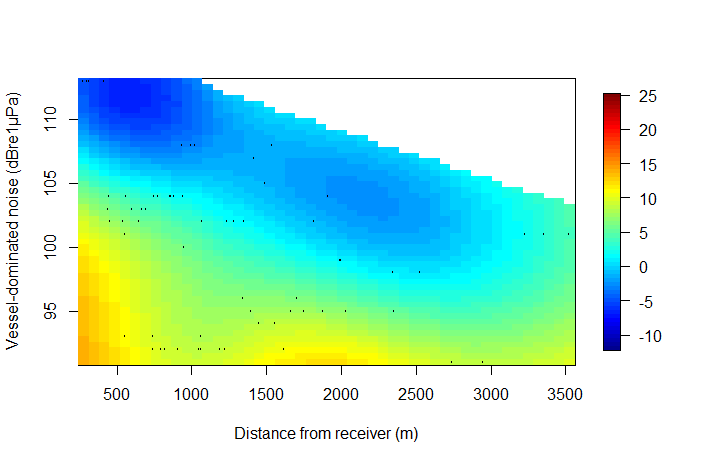


**b**

Distance from the signalling group (m)

Fig. S1. The relationship between the distance of the signalling group from the receiver (x axis; note the different scales), broadband noise levels (y axis; note the different scales), and measured *rSNR* of low-frequency (a) and high-frequency (b) vocal sounds. The representative frequency measures used were log F_Q3_ (set at 2.2 Hz; a) and log F_C10_ (set at 2.40 Hz; b) and time variable was T_Q3_ (set at 0.8 s and 0.3 s for a and b respectively) to control for the effects of frequency and duration. Data sourced from Dunlop 2018.


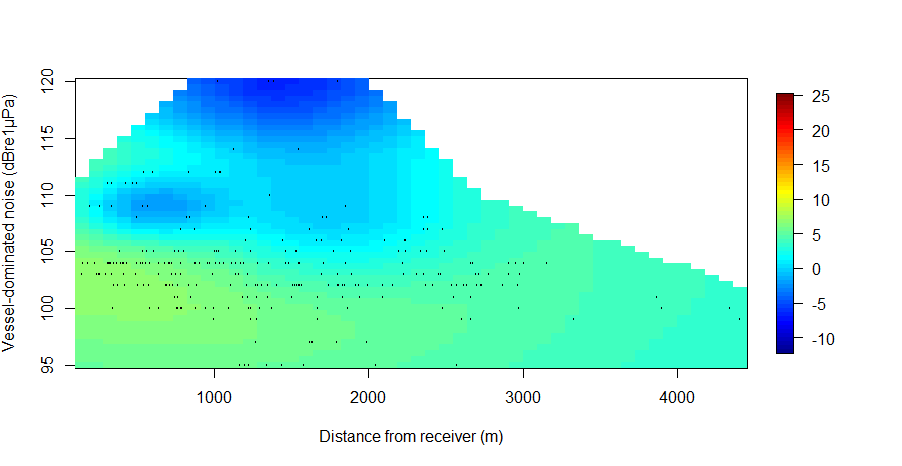

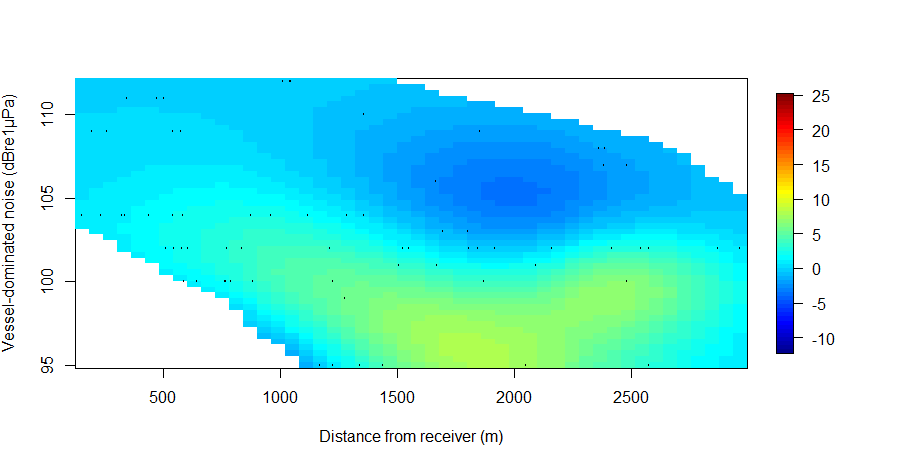


Distance from the signaling group (m)

Broadband vessel noise (dB *re* 1 µPa)

**a**

**b**

Fig. S2. The relationship between the distance of the signalling group from the receiver (x axis; note the different scales), broadband noise levels (y axis; note the different scales), and measured *rSNR* of low-frequency (a) and high-frequency (b) vocal sounds. The representative frequency measures used were log F_Q3_ (set at 2.2 Hz; a) and log F_C10_ (set at 2.40 Hz; b) and time variable was T_Q3_ (set at 0.8 s and 0.3 s for a and b respectively) to control for the effects of frequency and duration. Data sourced from Dunlop 2018*.*

**Behavioural analysis.** For every 10 minutes of the recording, the mean distance of each neighbour, being the nearest neighbour, 2^nd^ nearest neighbor, and so on for every group within a 5 km radius of the signaling group was measured. This gave between two and eight measured time periods per group, depending on the length of the recording, and between zero and six neighbours per time period. The separation distances of the neighbours and signalling groups (up to the 5^th^ nearest neighbour) are illustrated in Fig. S3.


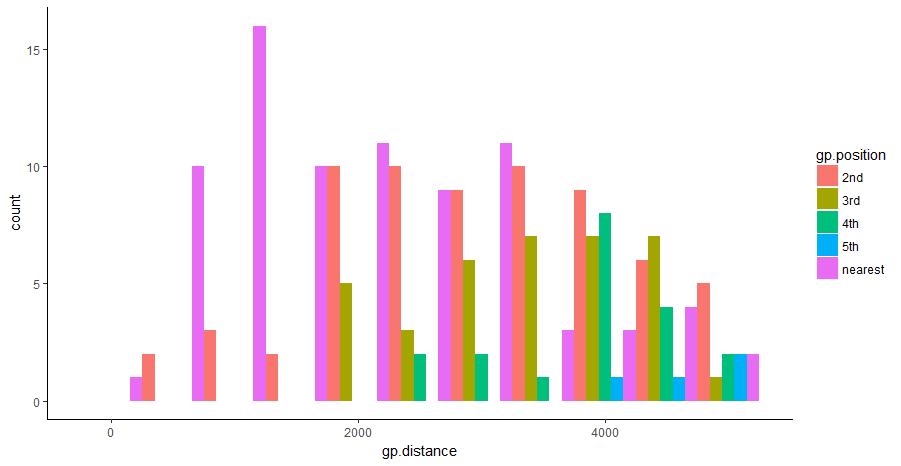


Distance from signalling group (m)

Count

neighbour position


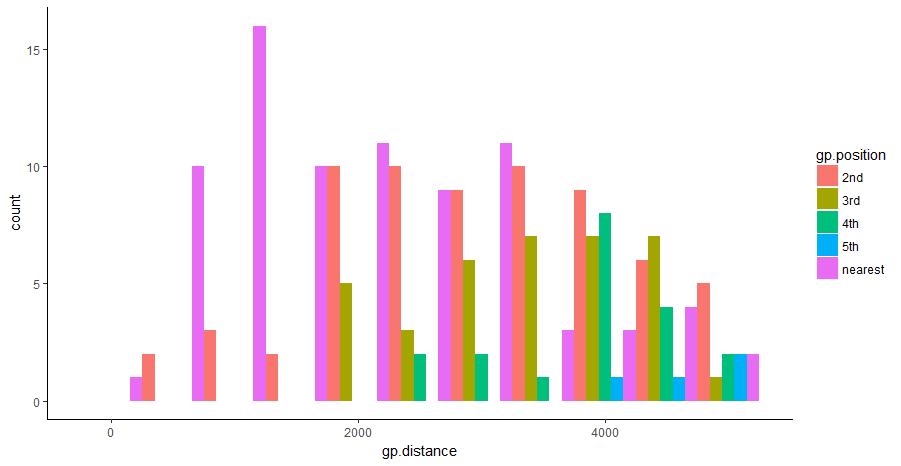


2^nd^ nearest

3^rd^ nearest

4^th^ nearest

nearest

5^th^ nearest

Fig. S3. The local social environment of signalling whales illustrating the distribution of distances of the nearest receiving neighbour, 2^nd^ nearest neighbour, and so on, from the signalling group counted every 10 minutes during the recording.

A linear model including neighbour distance from signalling group as the response variable and the interaction effect of noise source and neighbour position (nearest, 2^nd^….) was then ran to check that groups were not more widely dispersed from the signalling group for one or other of the noise environments. The two social environments (in wind and vessel noise) were not found to be significantly different.

Social behaviour was then quantified for each neighbour according to whether (1) or not (0) it interacted with the signalling group. An interaction was defined as being joined by, or joining, another group, or another group changing direction and approaching to within approximately 200 m of the signalling group. The first 10 minutes of each signalling group’s recording was annotated with the distance and position of each neighbour, and whether or not (1 or 0) the neighbour interacted with the signalling group at any time whilst the groups were visible in the survey area. This could have been when vocalisations from the group were no longer audible but the two groups (vocalising group and neighbouring group) were still visible to the land-based observers. If no interactions were sighted between the vocalising group and any of the neighbouring groups, the next 10 minutes of the recording was annotated as above, and so on, to give a distribution of neighbour distances from all non-interacting signalling groups.

If any of the neighbouring groups interacted with the signalling group, it either did so within the first 10 minutes, or if more than 1.5 km away, within a subsequent time bin whilst the signalling group was still visible. Usually, it was the nearest neighbour, so the interaction was sighted to have occurred within the first 10 minutes of the acoustic recording. In the latter scenario (neighbouring group more than 1.5 km away), the distance of the interacting neighbor would have been measured as getting progressively closer for each annotated time bin until they were sighted to have joined.

These two scenarios were dealt with differently. If a neighbour interacted with the signalling group within the first 10 minutes of the recording, subsequent 10 minute observations were annotated and used. This is because the social environment was different post-join in that the (still) signalling group was now comprised of new animals, and the distribution of the neighbouring groups would also have been different given the closest one had joined. If the neighbour did not interact within the first 10 minutes, but later on during observations, only the first 10 minutes was used in the analysis with subsequent time bins being removed until the two groups had joined. Here, the joining group was always sighted as approaching the signalling group but, due to the separation distance, took longer than 10 minutes to join. This gave the best estimate of the separation distance at which the two groups first began to approach each other. Removing subsequent time bins also removed repeated distance measures for the same interacting neighbour/signalling group pair. It should be noted that given the limitations of the visual observations, it cannot be confirmed that signalling groups and neighbours did not interact when out of view. However, these observations provide a record of group interactions within a defined study site.

**References**

Dunlop RA. 2018. The communication space of humpback whale social sounds in wind-dominated noise. *J Acoust Soc Amer* **144**(2):540-551.

Dunlop RA. The communication space of humpback whale social sounds in vessel noise. *Proceedings of Meetings on Acoustics,* in press, accepted 20/11/18.

Dunlop RA, Cato DH, Noad MJ, *et al.* 2013. Source levels of social sounds in migrating humpback whales (*Megaptera novaeangliae*). *J Acoust Soc Amer* **134**(1): 706-714.

Hardin JW. 2005. Generalized Estimating Equations (GEE). *Encyclopedia of Statistics in Behavioral Science.*

Højsgaard S, Halekoh U, and Yan J. 2006. The R Package geepack for Generalized Estimating Equations. *J Statistic Soft* **15**(2): 1-11.

Scott Hayward LAS, MacKenzie ML, Donovan CR, *et al.* 2014. Complex Region Spatial Smoother (CReSS). *J Computation Graph Stat* **23**(2): 340-360.

Walker, CG, Mackenzie ML, Donovan, CR, *et al.* **2011**. SALSA – a spatially adaptive local smoothing algorithm. *J Statistic Comp Sim* **81**(2).

Yan J, and Fine JP. 2004. Estimating Equations for Association Structures. *Stat in Med* **23**: 859-880.
